# Chemometric approach to find relationships between physiological elements and elements causing toxic effects in herb roots by ICP-MS

**DOI:** 10.1038/s41598-021-00019-w

**Published:** 2021-10-19

**Authors:** Adam Sajnóg, Elwira Koko, Dariusz Kayzer, Danuta Barałkiewicz

**Affiliations:** 1 Department of Trace Analysis, Faculty of Chemistry, Adam Mickiewicz University, Uniwersytetu Poznańskiego 8, 61-614 Poznań, Poland; 2 Department of Mathematical and Statistical Methods, Poznań University of Life Sciences, Wojska Polskiego 28, 60-637 Poznań, Poland

**Keywords:** Mass spectrometry, Statistics, Metals

## Abstract

In this paper 13 elements, both physiological and causing toxic effects, were determined by inductively coupled plasma mass spectrometry in roots of 26 species of herbs used in Traditional Chinese Medicine. The herbs were purchased from online shop in two batches 1 year apart to verify the variability of elemental content in time. The multivariate statistical methods—multiple regression, canonical variates and interaction effect analysis—were applied to interpret the data and to show the relationships between elements and two batches of herb roots. The maximum permissible concentration of Cd (0.3 mg kg^−1^) was exceeded in 7 herb roots which makes 13% of all specimens. The multiple regression analysis revealed the significant relationships between elements: Mg with Sr; V with Pb, As and Ba; Mn with Pb; Fe with As and Ba; Co with Ni and Sr, Cu with Pb, Cd and As; Zn with Pb, Cd, As and Ba. The canonical variates analysis showed that the statistical inference should not be based solely on the type of herb or number of batch because of the underlying interaction effects between those two variables that may be a source of variability of the content of determined elements.

## Introduction

Herbs of Chinese origin are used as alternative medicines, dietary supplements and herbal infusions and are used not only in China, which has been based on traditional medicinal practice (TCM—Traditional Chinese Medicine) since ancient times, but also in Europe and North America^1,2^. They are considered an important source of vitamins and plant proteins, thus supporting immunity^3^. Their unskillful use or combination with other drugs and herbs can lead to dangerous side effects. European pharmaceutical law cannot be enforced on herbs to increase control and safety because current regulations do not treat herbs as drugs but as food^4^. In addition, the basis of European herbal preparations is usually one active substance, and in Chinese blends there are several of them and each requires appropriate characteristics and tests. The World Health Organization WHO has developed a strategy for 2014–2023 that supports the safe use of TCM by regulating practices and products^5^. The European Pharmacopoeia^6^ mentions tests for heavy metals, mycotoxins, pesticides and microbial contamination on herbal drugs and sets limits for Cd (1 mg kg^−1^), Pb (5 mg kg^−1^) and Hg (0.1 mg kg^−1^). The interest in the elemental composition of herbs increases with the development of nutrition and biochemical research. Examination of herbs to determine the content of physiological elements and elements causing toxic effects will allow to assess the degree of poisoning caused by the consumption of Chinese herbs. China is struggling with one of its most serious environmental problems, namely environmental pollution from rapid urbanization and intensive agricultural and industrial production which has poisoned soils with heavy metals^3,7^. As a consequence, the toxic elements present in the soil are taken up by plants grown in contaminated areas^8^. The content of elements, both physiological and causing toxic effect, is highly dependent on soil type, irrigation, season, the type and part of the plant and the stage of ripening^9^. Weak physiological barrier of plants against the uptake of elements causing toxic effect and defense mechanisms include: (a) formation of intracellular protein chelates (phytochelatin), (b) active export, (c) impeded transport through the cell membrane, (d) immobilization in the cell wall, especially by pectins, (e) chelation by molecules from internal metabolism, and then the formation of a vacuole in which unnecessary metabolic products are stored^10^. The exposure to stress caused by toxic factors does not eliminate them completely from the biological system, so there is still a risk of including them in the food chain^11^. Trace elements exhibit various geochemical properties. Chemical forms in which they occur in soil determine their mobility and the degree of absorption by plants, and so Cr, Ni and Pb are the least mobile, while Cd, Zn and Mo are the most mobile^12^. Trace elements are key components of enzymes that perform important biological functions^13^. The literature shows that toxic elements such as Cd and Pb accumulate mainly in plant roots^14,15^. The passive or active mechanism of uptake of elements from the soil depends on the physicochemical conditions of the rhizosphere. After collection, elements can accumulate in the roots, for example: Cd, Co, Cu, Cr, Pb, medium-mobile elements such as Mn, Ni, Zn can move to the stem, and Se is easily transported to the above-ground parts of the plant. Trace elements, both physiological and causing toxic effect, occurring in excess have a detrimental effect on various processes in plant cells^16^. It has been shown that there are correlations between elements in living organisms. They can be antagonistic, synergistic, both simultaneously, or show no correlation at all^12,17^.

Recent studies indicate that the contamination of herbs with trace elements is a problem for consumers. The greatest threats are Pb, Cd, As, Ni and Cr, as they have a high carcinogenic potential^3^. Element toxicity may vary depending on the dose, administration route, chemical form of element and time of exposure. The toxic effects of Pb include degeneration of nervous system, nephropathy and anemia. Chronic exposition to Pb may lead to accumulation in bones and teeth. Cadmium may severely damage kidneys, liver and lungs and can accumulate in human body with very long half-life (ca. 20 years). The chronic effects of Cd include kidney disfunctions, bone fractures, hypertension and pulmonary lesions. The toxic effects of As are widely recognized, including damage to the skin, kidneys, bladder and gastro-intestinal organs. However, As does not usually accumulate in the body and is expelled within days, depending on dose and form^18^. The elements Pb, Cd and As have detrimental effect on fertility and fetus health. Several cases of lead poisoning from the consumption of Chinese herbs in the use of TCM have been reported in the past 20 years^19–22^. There are also reports of Hg and As poisoning^23^ and allergic reactions, as most herbal products do not require proof of efficacy and safety^24^. A single consumption of herbal products does not cause negative health effects. However, consuming them for a long time and in large amounts poses the risk of poisoning which was a premise of this study^13^.

The most common method of preparation of plant samples for analysis is wet digestion with the use of nitric acid and hydrogen peroxide with microwaves^25–27^. Methods such as atomic absorption spectrometry (AAS)^13,25^, inductively coupled plasma mass spectrometry (ICP-MS) with a single quadrupole^13,14,23,26,28^ and triple quadrupole^29^, inductively coupled plasma optical emission spectrometry (ICP-OES)^30,31^ and instrumental neutron activation analysis (INAA)^30,32^ are used to determine elements in herbs and similar plant material. ICP-MS is a technique that allows for multielemental analysis with very low detection limits and linear range of over nine orders of magnitude which makes it suitable to determine most metallic elements at ng L^−1^ and µg L^−1^ to hundreds mg L^−1^ levels in the single analytical run.

The aim of this study is to examine the content of elements, both physiological and causing toxic effects, in the roots of herbs of Chinese origin used in TCM. A multielemental analytical procedure was developed, employing ICP-MS technique with spectral and non-spectral interference removal methods. In order to obtain the reliable measurement results the analytical procedure was validated, characteristic parameters have been determined and traceability is ensured through the use of matrix-matched certified reference materials (CRM). For interpretation of data and finding relationships between elements the multivariate statistical methods such as multiple regression analysis, canonical variates analysis with interaction effects were applied.

## Materials and methods

### Reagents and labware

The multielemental standard solution 3 (Perkin-Elmer, USA) containing all analytes in concentration 10 mg L^−1^ and single standard stock solution of Mg, Mn, Fe, Zn, Sr and Ba (Merck) with concentration 1000 mg L^−1^ were used to prepare calibration standards by a series of dilutions. Nitric acid (65%, Suprapur, Merck, Germany) was used to acidify standard solutions and as a digestion reagent. Hydrogen peroxide (30%, Suprapur, Merck, Germany) was used as a digestion reagent. All dilutions were carried out with Milli-Q water (Direct-Q 3 UV, Merck, Germany). 25 mL polyethylene volumetric flasks were used to prepare calibration standards. 50 mL polypropylene Falcon tubes were used to dilute samples of herbs after digestion. All labware was soaked in 1% nitric acid for 24 h and flushed with Milli-Q water before use.

### Research material

The research material was the dried herbal roots used in TCM. The Latin and Chinese names of herbs with the recommended dose are gathered in Table 1. The roots were purchased as two batches, 1 year apart—in 2020 and 2021—from an online shop specializing in TCM; all roots originated from China. The roots were sold as dried material in fragments of varying sizes (from several mm to 2 cm of longer side) and were delivered and stored in closed zip-lock bags in dry, dark place until the analysis. The preliminary preparation of herbs consisted of the gentle washing with Milli-Q water to clean the surface of roots from dust and air-drying for 24 h in 40 °C in the laboratory dryer. The dried roots were then ground in a ball mill (Mini-Mill Pulverisette 23, FRITSCH, Germany). The handling and experiments on plant material in this study were performed in accordance with relevant institutional, national and international guidelines and legislation.

**Table 1:** Latin and Chinese names of investigated herbs and the recommended dose for preparation of a single infusion for medicinal purposes.

Number of herb	Latin name	Chinese name	Recommended dose (g)	Examples of applications
1	*Achyranthis*	Niu Xi	9–15	Cardiovascular, gynecological diseases, arthritis
2	*Angelicae* *dahuricae*	Bai Zhi	3–9	Colds, headaches
3	*Angelicae* *pubescentis*	Du Huo	3–9	Rheumatic arthritis, headache
4	*Angelicae* *sinensis*	Dang Gui Wei	3–15	Constipation, boosts immunity
5	*Asparagi*	Tian Men Dong	6–15	Asthma, constipation, high blood pressure
6	*Astragali*	Huang Qi	9–30	Cold, boosts immunity, high blood pressure, diabetes
7	*Codonopsis*	Dang Shen	9–30	Boosts immunity, anorexia, chronic diarrhea, asthma
8	*Dipsaci*	Xu Duan	6–21	Bones and joints diseases
9	*Gentianae* *macrophyllae*	Qin Jiao	4.5–12	Stimulates digestion, high blood pressure, fever
10	*Glehniae*	Bei Sha Shen	9–15	Cough, fevers
11	*Isatidis*	Ban Lan Gen	15–30	Influenza, hepatitis
12	*Morindae* *officinalis*	Ba Ji Tian	6–15	Urinary system diseases, infertility
13	*Notopterygii*	Qiang Huo	6–15	Cold, fever, body pains
14	*Paeoniae* *alba*	Bai Shao	6–15	Relives pain, rheumatoid arthritis, hepatitis
15	*Paeoniae* *rubra*	Chi Shao	4.5–9	Relives pain, menstrual disturbances
16	*Platycodi*	Jie Geng	3–9	Fever, cough, asthma
17	*Polygalae*	Yuan Zhi	3–9	Improves cognitive functions, cough
18	*Pseudostellariae*	Tai Zi Shen	9–30	Fatigue, spleen diseases, cough
19	*Puerariae*	Ge Gen	6–12	Diarrhea, cardiovascular diseases, fever
20	*Pulsatillae* *chinensis*	Bai Tou Weng	6–15	Diarrhea, inflammations, microbial diseases
21	*Rhei*	Da Huang	3–12	Digestive diseases
22	*Saposhnikoviae*	Fang Feng	3–9	Arthritis, pain, inflammations
23	*Scrophulariae*	Xuan Shen	9–30	Cardiovascular diseases, fever
24	*Stephaniae* *tetrandrae*	Fang Ji	3–9	Rheumatism, relives pain, promotes diuresis
25	*Trichosanthis*	Tian Hua Fen	9–15	Cardiovascular diseases, angina
26	*Vladimiriae* *soulier*	Chuan Mu Xiang	1.5–9	Digestive diseases, relives pain

### Sample preparation

Acidic digestion in a closed system with microwaves was carried out. About 0.3 g of the ground sample, 3 mL 65% nitric acid and 1 mL 30% hydrogen peroxide were used for digestion in quartz vessels. The whole process was carried out in the microwave digestion system (Ethos One, Milestone Srl, Italy) in 3 stages: 20 min ramp time to 200 °C, 30 min at 200 °C and cooling down. The final step in sample preparation was the quantitative transfer of the digested sample to Falcon tubes and an 80-fold dilution with Milli-Q water.

### Analytical method

Calibration solutions were prepared by appropriately diluting a multi-element calibration standard and single element standards. The calibration standards concentration varied depending on the analyte. For all analyzed elements the following concentrations of calibration solutions were prepared (in µg L^−1^): 0.050, 0.10, 0.50, 1.0, 5.0, 10, 50. For elements expected to be more abundant in the roots of herbs the single element stock solutions were used to prepare solutions with higher concentration (in µg L^−1^): 200 for Ni and Cu; 500, 1000, 2000 for Mn, Zn, Sr and Ba; 5000 for Fe; 20,000 for Mg.

The multielement analysis of roots of herbs after digestion was performed in ICP-MS (Agilent 7700x, USA) equipped with octopole reaction system (ORS) working in no-gas and helium mode for the reduction of spectral interferences. Non-spectral and matrix interferences were minimized by diluting the samples and using an internal standard solution containing 10 µg L^−1^ Rh, introduced in parallel with the analyzed solution via T-piece. To ensure the accuracy and correctness of the analytical procedure three CRMs were used: Mixed Polish Herbs (INCT-MPH-2, Institute of Nuclear Chemistry and Technology, Warsaw, Poland), Trace Elements in Spinach Leaves (1515, NIST, USA), Apple Leaves (1570a, NIST, USA). The CRMs were digested in triplicates according to the procedure described in sample preparation chapter. Ten procedural blanks were obtained in the same procedure from the digestion reagents in the digestion vessel. The average value measured for procedural blanks was subtracted from the concentrations measured for digested roots. After calibration the measurement control was conducted through the analysis of standard solutions and CRMs after every ten herb samples.

The operating conditions of ICP-MS are presented in Table 2.

**Table 2:** Operating instrumental conditions of ICP-MS.

Instrument	Agilent 7700x	
Nebulizer/spray chamber	Seaspray 0.4 mL min^−1^/quartz Scott double pass	
Nebulizer gas flow (L min^−1^)	1.0	
Auxiliary gas flow (L min^−1^)	0.9	
Plasma gas flow (L min^−1^)	15	
RF Power (W)	1550	
Peristaltic pump speed (rps)	0.1	
Lens Parameters (V)	Extract1: 0.0 Extract2: − 160 Omega lens: 7.6 Omega bias: − 70	Cell entrance: − 30 Cell exit: − 50 Deflect: 14 Plate bias: − 40
He flow rate (mL min^−1^)	4.3	
Integration time per m/z (s)	0.1	
Replicates	3	
Sweeps per replicate	100	

### Statistical and chemometric evaluation

In order to evaluate the relationships between elements in herb roots the multivariate methods were used to analyze the results of the measurement. Canonical variate coordinates are directions in multivariate space that maximally separate the predefined groups of the analyzed dataset^33,34^. Canonical variate analysis (CVA) and modifications thereof are widely used in various scientific fields, not only to study the content of elements^35^, but also environmental^36^, agriculture and biological sciences^37^ and economics^38^. Additionally, the CVA, similar to principal component analysis, also showed relations between selected experimental objects and the variables that describe them^39,40^. In this case, the experimental object was defined as the values of content of elements for the particular herb root in the individual batch. This method was chosen because we consider the data sets coming from the model classified according to two sources of variability: batch and herb root. The method is based on studying the model for two-way classification^41^ by analyzing the interaction in herb root by batch and interaction effects. To compare experimental objects and interaction effects, the Mahalanobis distances^39^ were calculated based on the results of element content in roots. The differences between herb roots may be either due to the fact the herb root factor causes some interaction with the batch factor or because there are some significant main effects of the former factor, or due to both^41^.

The multiple regression method was used, and the parameters of equations which address separately the physiological elements (Mg, V, Mn, Fe, Co, Cu, Zn) were estimated in order to determine the strength and direction of impact of elements causing toxic effect on physiological elements in herb roots. The relationships between the content of physiological elements in herb roots and the analyzed elements causing toxic effects were determined using the multiple linear regression equation:

$$\left[y\right]={\beta }_{0}+{\beta }_{1}\left[Pb\right]+{\beta }_{2}\left[Cd\right]+{\beta }_{3}\left[As\right]+{\beta }_{4}\left[Ni\right]+{\beta }_{5}\left[Ba\right]+{\beta }_{6}\left[Sr\right]+e$$

where $\left[y\right]$—content od physiological element (in mg kg^−1^) in herb roots, ${\beta }_{i}$—regression coefficient, $e$—random error.

Multiple linear regression and stepwise regression with backward elimination were performed using the statistical software Statistica 13 (Statsoft, Poland).

## Results and discussion

The proper conclusions drawn from the experimental data require that the measurements were performed accurately. In order to verify that the developed analytical procedure is fit for intended purpose it was validated by analyzing series of calibration standards, blank samples and matrix-matching CRMs, and by estimating the parameters characterizing the analytical method: working range, linearity, limits of detection and quantification, precision and trueness. After validation of the analytical procedure the elements in samples of herb roots were determined and the obtained results were evaluated with the basic statistics, as well as multivariate statistical methods.

### Method validation

Obtained values with validation parameters are presented in Table 3. Calibration curves for elements determined by ICP-MS were in the range defined by the estimated limit of quantification and the standard with highest concentration. The lowest upper limits of working range equal 50 µg L^−1^ have V, Co, As, Cd and Pb. The standards with highest upper limits of working range equal 20,000 µg L^−1^ were prepared for Mg, 5000 µg L^−1^ for Fe and 2000 µg L^−1^ for Mn and Zn. Calibration curves for all elements were linear over the entire concentration range which resulted in high correlation coefficients > 0.9998 with exception of 0.9991 for Mg.

**Table 3:** The validation parameters of the developed analytical procedure for determination of 13 elements in roots of herbs.

	Mg	V	Mn	Fe	Co	Ni	Cu	Zn	As	Sr	Cd	Ba	Pb
Working range [µg L^−1^]	1.1–20,000	0.027–50	0.16–2000	1.4–5000	0.0076–50	0.12–200	0.12–200	1.4–2000	0.055–50	0.14–1000	0.033–50	0.16–1000	0.030–50
Linearity *R*	0.9991	0.9999	0.9998	0.9998	0.9999	0.9999	0.9998	0.9998	0.9999	0.9998	0.9999	0.9999	0.9999
IDL [µg L^−1^]	0.31	0.0088	0.055	0.46	0.0025	0.040	0.041	0.48	0.018	0.049	0.011	0.053	0.0099
IQL [µg L^−1^]	0.92	0.027	0.16	1.4	0.0076	0.12	0.12	1.4	0.055	0.15	0.033	0.16	0.030
MDL [µg g^−1^]	3.2	0.0097	0.051	0.37	0.0041	0.059	0.038	0.35	0.015	0.063	0.0072	0.079	0.0087
**Repeatability as CV [%]**													
Low conc	15	6.1	26	20	22	20	7.0	12	65	14	17	17	26
Medium conc	7.7	4.2	3.1	6.1	3.0	4.4	1.3	5.3	4.3	4.6	4.0	5.5	3.8
High conc	3.5	1.0	1.5	1.5	1.3	1.5	0.68	2.4	2.0	2.0	1.7	2.2	1.5
**Intermediate precision as CV [%]**													
Low conc	20	21	28	34	15	6.8	7.2	23	31	21	19	29	15
Medium conc	5.3	8.0	8.3	13	6.7	6.7	6.5	10	7.9	12	7.7	7.5	6.8
High conc	4.2	5.3	4.2	6.0	4.7	5.7	6.2	4.3	5.7	5.0	5.6	4.9	3.8
**Trueness as recovery ± SD [%]** *p* value of Student’s *t* test													
Spinach leaves 1570a	^#^83 ± 9 0.407	83 ± 7 0.109	75 ± 8 0.053	*	83 ± 5 0.463	81 ± 9 0.122	80 ± 7 0.100	88 ± 5 0.194	106 ± 16 0.768	76 ± 8 0.062	79 ± 6 0.006	*	^#^81 ± 6 0.366
Apple leaves 1515	89 ± 12 0.422	84 ± 11 0.118	82 ± 12 0.212	95 ± 6 0.416	95 ± 12 0.799	91 ± 11 0.477	89 ± 11 0.372	104 ± 10 0.763	*	85 ± 12 0.302	113 ± 9 0.680	85 ± 11 0.263	84 ± 11 0.102
Mixed polish herbs INCT-MPH-2	84 ± 5 0.062	80 ± 5 0.084	79 ± 8 0.092	^#^84 ± 9 0.370	80 ± 4 0.075	76 ± 9 0.104	84 ± 3 0.068	88 ± 9 0.278	89 ± 9 0.391	82 ± 4 0.061	83 ± 6 0.734	79 ± 5 0.062	105 ± 4 0.463

The IDL and IQL refer to the solution and MDL refers to the dry mass of roots. The low and high concentration refers to the standard solution with concentration close to the low limit of working range and the highest concentration standard, respectively. The medium concentration is (in µg L^−1^) 1.0 for V, Co, As, Cd and Pb, 10.0 for Ni and Cu, 100 for Mn, Fe, Zn, Sr and Ba, and 1000 for Mg. The Student’s *p* value refer to the probability of statistical significance between measured and certified value in CRM.

*Element not stated in certificate;

^#^Element with informative value.

Instrumental detection limits (IDL) were calculated as three standard deviations (SD) of the calibration blank samples. The lowest values of IDL were 0.0025 and 0.0088 µg L^−1^ for Co and V, respectively, and the highest: 0.48 and 0.46 µg L^−1^ for Zn and Fe, respectively. Method detection limits (MDL) were calculated as three SD from the procedural blank samples. The lowest values of MDL were 0.010 and 0.018 µg L^−1^ for Co and Cd, respectively, and the highest: 8.1 and 0.91 µg L^−1^ for Mg and Fe, respectively. Instrumental quantification limits (IQL) were calculated as three times IDL, with the lowest values: 0.0076 and 0.027 µg L^−1^ for Co and V, respectively, and the highest: 1.4 µg L^−1^ for Fe and Zn. In all analyzed herbs the obtained concentrations are above the detection limit.

The precision was calculated as the relative standard deviation of three repetitions of calibration standards on three concentration levels and expressed in % as coefficient of variation (CV) with distinction to short-term precision measured in a single analytical run (repeatability) and long-term measured in the span of 3 days (intermediate precision). Repeatability values for all analyzed elements were in the range from 0.68% for Cu to 65% for As, and the intermediate precision values were usually higher—in the range from 4.2% for Mg and Mn to 34% for Fe.

The trueness was estimated by analyzing three CRMs which were subjected to the same digestion procedure as herb samples in three independent replicates of 0.3 g each. The applied CRMs were NIST SRM 1570a Trace Elements in Spinach Leaves, NIST SRM 1515 Apple Leaves and INCT-MPH-2 Mixed Polish Herbs. The obtained recoveries of certified concentrations are in the range from (75 ± 8%) for Mn to (113 ± 9%) for Cd. Other CRMs used in studies on elemental content of herbs found in literature are GBW 07604 (poplar leaves)^17^, IAEA-336 (lichen)^32^, NIST SRM 1570a (trace elements in spinach leaves)^28,30,32^, NCS DC73349 (trace elements in bush branches and leaves)^42^, Japan CRM NIES no. 7 (tea leaves)^31^. A review article on determination of elements in herbs and CRMs used for method validation was published by Pohl et al.^43^.

### Determination of elements in herbs

The elements were grouped depending on their effect on the human health: physiological elements (Mg, V, Fe, Mn, Co, Cu, Zn) and the second group contains elements that are causing toxic effects (Pb, Cd, As, Ni, Ba, Sr). Elements causing toxic effect have negative effect on health even in low concentrations which include Pb, Cd, As Ni, while Sr and Ba are consider not toxic in the concentrations usually find in food, but may induce toxic effects in higher doses. It is worth noting that certain elements may have detrimentally different toxicity depending on the speciation form, for example BaCl_2_ and BaSO_4_. However, the speciation analysis is not in scope of this study and only the general content of elements is discussed.

The results of determination of 13 elements in 26 herbs each analyzed in two batches purchased 1 year apart ($n=52$) are presented in Table 4. The two batches of herbs are described as “A” and “B”, referring to samples obtained in 2020 and 2021, respectively. The basic descriptive statistics of the measured contents of elements in herb roots are gathered in Table 5. The obtained results show considerable variation among elements and tested herbs. The largest values were measured for Mg with the maximum content of 6947 mg kg^−1^ in herb 19A (*Puerariae*). The lowest values were measured for Cd with the minimum content of 0.0040 mg kg^−1^ in herb 25B (*Trichosanthis*). Relatively, the narrowest range of content values for all herbs characterizes elements: Mg, Zn and Cu, and the broadest range have Pb and Cd. The average contents of elements causing toxic effects measured for all herbs are in the order: Cd < As < Pb < Ni < Sr < Ba.

**Table 4:** The measurement results of 13 elements in 26 herb roots each analyzed in two batches purchased 1 year apart ($n=52$).

Herb	Mg	V	Mn	Fe	Co	Ni	Cu	Zn	As	Sr	Cd	Ba	Pb
1A	2559.199 ± 30.457	0.257 ± 0.002	29.540 ± 0.482	278.178 ± 2.685	0.131 ± 0.002	0.346 ± 0.005	3.430 ± 0.022	12.286 ± 0.182	0.076 ± 0.002	17.777 ± 0.329	0.026 ± 0.002	4.375 ± 0.075	0.059 ± 0.001
1B	1756.890 ± 44.862	0.836 ± 0.039	34.905 ± 0.237	325.478 ± 5.730	0.270 ± 0.007	0.913 ± 0.034	5.784 ± 0.157	15.681 ± 0.497	0.246 ± 0.014	29.991 ± 1.267	0.124 ± 0.018	6.898 ± 0.590	0.303 ± 0.025
2A	1623.176 ± 54.412	0.284 ± 0.011	12.658 ± 0.630	254.531 ± 11.846	0.107 ± 0.004	0.331 ± 0.019	6.057 ± 0.254	11.837 ± 0.334	0.079 ± 0.010	21.249 ± 0.977	0.011 ± 0.005	9.217 ± 0.356	0.059 ± 0.002
2B	1327.251 ± 20.732	0.907 ± 0.029	20.071 ± 0.128	372.772 ± 2.394	0.235 ± 0.008	0.785 ± 0.033	7.796 ± 0.089	13.518 ± 0.216	0.176 ± 0.015	20.821 ± 0.142	0.021 ± 0.005	10.893 ± 0.609	0.259 ± 0.022
3A	2695.134 ± 30.960	0.462 ± 0.014	188.405 ± 2.896	408.833 ± 2.181	0.576 ± 0.014	1.694 ± 0.051	13.413 ± 0.106	22.825 ± 0.345	0.099 ± 0.003	32.096 ± 0.974	0.559 ± 0.079	46.042 ± 0.953	0.954 ± 0.026
3B	1929.060 ± 12.612	1.501 ± 0.061	41.497 ± 0.701	742.310 ± 16.790	0.322 ± 0.010	0.988 ± 0.035	13.321 ± 0.206	18.271 ± 0.656	0.356 ± 0.032	16.742 ± 0.183	0.070 ± 0.011	40.154 ± 1.124	0.383 ± 0.014
4A	1652.102 ± 19.950	1.724 ± 0.019	20.294 ± 0.237	631.181 ± 6.622	0.241 ± 0.004	0.659 ± 0.015	5.840 ± 0.093	16.760 ± 0.178	0.438 ± 0.012	18.640 ± 0.275	0.017 ± 0.002	9.156 ± 0.129	0.348 ± 0.006
4B	1976.210 ± 45.357	2.299 ± 0.074	24.188 ± 0.433	1020.412 ± 17.347	0.365 ± 0.006	1.126 ± 0.046	8.109 ± 0.160	17.730 ± 0.321	0.628 ± 0.050	15.756 ± 0.297	0.048 ± 0.005	14.413 ± 0.698	0.629 ± 0.031
5A	1084.852 ± 50.113	0.063 ± 0.007	16.115 ± 0.770	84.931 ± 3.375	0.451 ± 0.016	0.628 ± 0.019	5.722 ± 0.242	14.160 ± 0.739	0.025 ± 0.001	7.898 ± 0.486	0.045 ± 0.002	7.391 ± 0.199	0.051 ± 0.006
5B	643.324 ± 15.900	0.114 ± 0.007	20.769 ± 0.524	66.641 ± 1.329	0.983 ± 0.021	1.810 ± 0.044	5.852 ± 0.104	15.141 ± 0.423	0.044 ± 0.006	5.407 ± 0.147	0.142 ± 0.003	8.103 ± 0.090	0.134 ± 0.004
6A	2707.653 ± 96.581	0.715 ± 0.012	20.734 ± 0.446	564.071 ± 15.101	0.171 ± 0.009	2.082 ± 0.046	7.039 ± 0.238	24.365 ± 0.711	0.237 ± 0.010	14.551 ± 0.485	0.023 ± 0.007	6.592 ± 0.172	0.128 ± 0.004
6B	1334.043 ± 38.907	0.606 ± 0.014	14.762 ± 0.213	251.588 ± 1.892	0.132 ± 0.005	1.798 ± 0.021	6.305 ± 0.055	29.186 ± 0.157	0.188 ± 0.012	13.114 ± 0.276	0.022 ± 0.004	5.445 ± 0.470	0.154 ± 0.031
7A	1240.004 ± 5.298	0.810 ± 0.008	23.946 ± 0.459	322.469 ± 1.668	0.182 ± 0.004	1.016 ± 0.008	3.554 ± 0.025	19.877 ± 0.533	0.217 ± 0.011	22.638 ± 0.195	0.052 ± 0.008	67.313 ± 0.693	0.181 ± 0.004
7B	1955.191 ± 35.020	3.635 ± 0.196	73.343 ± 1.624	1537.872 ± 23.736	0.721 ± 0.026	2.936 ± 0.070	8.943 ± 0.180	118.855 ± 1.305	0.865 ± 0.109	55.461 ± 0.709	0.079 ± 0.010	230.716 ± 2.435	1.169 ± 0.117
8A	5103.323 ± 31.804	2.194 ± 0.015	107.258 ± 1.258	1200.745 ± 11.335	0.702 ± 0.012	2.121 ± 0.039	6.297 ± 0.010	41.900 ± 0.122	0.291 ± 0.027	60.212 ± 0.982	0.311 ± 0.011	140.560 ± 0.788	1.039 ± 0.018
8B	3009.020 ± 55.301	2.596 ± 0.212	83.080 ± 1.724	978.995 ± 15.502	0.809 ± 0.017	3.085 ± 0.039	10.187 ± 0.117	33.828 ± 1.021	0.362 ± 0.052	39.186 ± 0.964	0.663 ± 0.063	116.455 ± 3.836	1.449 ± 0.060
9A	797.124 ± 16.342	0.934 ± 0.011	14.189 ± 0.213	345.919 ± 2.409	0.216 ± 0.005	1.219 ± 0.019	6.721 ± 0.055	25.395 ± 0.209	0.184 ± 0.007	28.483 ± 0.660	0.037 ± 0.002	30.911 ± 0.689	0.298 ± 0.008
9B	826.215 ± 28.683	0.619 ± 0.028	11.778 ± 0.623	252.871 ± 7.882	0.150 ± 0.013	1.420 ± 0.057	6.157 ± 0.251	29.397 ± 0.623	0.125 ± 0.034	24.629 ± 0.532	0.064 ± 0.016	32.900 ± 1.785	0.266 ± 0.021
10A	1415.922 ± 82.954	0.439 ± 0.046	8.774 ± 0.695	147.540 ± 7.290	0.172 ± 0.011	0.545 ± 0.019	8.900 ± 0.493	16.850 ± 1.021	0.117 ± 0.010	10.792 ± 0.502	0.085 ± 0.005	19.387 ± 1.274	0.199 ± 0.015
10B	1356.942 ± 22.187	0.427 ± 0.014	6.503 ± 0.237	191.391 ± 3.657	0.151 ± 0.003	0.586 ± 0.017	8.958 ± 0.190	13.007 ± 0.336	0.083 ± 0.014	9.633 ± 0.167	0.059 ± 0.005	13.792 ± 0.089	0.124 ± 0.008
11A	3212.679 ± 39.687	0.566 ± 0.006	16.521 ± 0.538	436.339 ± 8.525	0.117 ± 0.006	0.589 ± 0.036	2.951 ± 0.095	25.631 ± 0.895	0.248 ± 0.054	96.662 ± 2.138	0.061 ± 0.005	10.831 ± 0.209	0.096 ± 0.007
11B	1157.541 ± 15.575	0.380 ± 0.007	9.512 ± 0.085	192.262 ± 2.780	0.088 ± 0.005	0.347 ± 0.007	1.616 ± 0.043	23.691 ± 0.478	0.123 ± 0.006	34.139 ± 0.502	0.063 ± 0.005	8.211 ± 0.134	0.108 ± 0.013
12A	806.960 ± 2.704	0.862 ± 0.029	474.823 ± 0.993	455.102 ± 5.933	0.215 ± 0.009	0.409 ± 0.001	5.809 ± 0.161	27.892 ± 0.749	0.214 ± 0.005	31.041 ± 0.224	0.155 ± 0.007	18.103 ± 0.437	9.062 ± 0.091
12B	1041.220 ± 29.760	1.415 ± 0.133	238.478 ± 4.887	813.701 ± 20.382	0.534 ± 0.015	0.746 ± 0.009	6.501 ± 0.170	30.946 ± 0.518	0.486 ± 0.006	19.716 ± 0.544	0.184 ± 0.026	29.877 ± 0.942	7.719 ± 0.204
13A	2596.411 ± 86.390	1.431 ± 0.035	61.098 ± 1.357	551.466 ± 9.620	0.466 ± 0.013	1.472 ± 0.034	9.164 ± 0.270	30.138 ± 0.473	0.440 ± 0.011	41.730 ± 0.959	0.130 ± 0.015	20.795 ± 0.442	0.577 ± 0.017
13B	2691.208 ± 35.192	1.960 ± 0.091	51.396 ± 0.934	804.680 ± 16.746	0.462 ± 0.008	1.937 ± 0.051	8.751 ± 0.167	28.182 ± 0.145	0.680 ± 0.042	39.237 ± 1.266	0.210 ± 0.009	24.892 ± 1.516	0.948 ± 0.026
14A	1168.082 ± 8.373	0.047 ± 0.003	19.651 ± 0.278	19.135 ± 0.143	0.057 ± 0.003	0.860 ± 0.006	5.117 ± 0.027	17.622 ± 0.217	0.028 ± 0.003	77.189 ± 2.626	0.038 ± 0.004	10.693 ± 0.412	0.021 ± 0.002
14B	825.792 ± 22.102	0.085 ± 0.016	9.316 ± 0.266	25.005 ± 0.657	0.052 ± 0.001	0.470 ± 0.016	4.787 ± 0.129	18.004 ± 0.551	0.036 ± 0.008	50.577 ± 1.380	0.052 ± 0.007	7.003 ± 0.339	0.052 ± 0.009
15A	4210.767 ± 82.060	0.545 ± 0.016	12.998 ± 0.143	342.052 ± 6.868	0.091 ± 0.003	2.302 ± 0.044	2.783 ± 0.033	29.998 ± 0.266	0.104 ± 0.020	203.974 ± 3.985	0.044 ± 0.003	56.031 ± 1.134	0.110 ± 0.002
15B	1944.009 ± 36.335	0.423 ± 0.103	10.774 ± 0.174	196.618 ± 4.644	0.103 ± 0.003	2.969 ± 0.052	3.273 ± 0.089	28.348 ± 0.826	0.095 ± 0.017	261.375 ± 4.243	0.069 ± 0.007	51.121 ± 2.177	0.159 ± 0.022
16A	2156.584 ± 32.968	0.280 ± 0.016	11.078 ± 0.232	182.309 ± 1.347	0.064 ± 0.003	0.423 ± 0.016	3.614 ± 0.090	17.390 ± 0.178	0.073 ± 0.027	27.211 ± 0.274	0.069 ± 0.011	8.991 ± 0.081	0.058 ± 0.002
16B	1522.359 ± 21.819	0.227 ± 0.030	9.850 ± 0.211	102.531 ± 1.299	0.050 ± 0.001	0.707 ± 0.010	3.755 ± 0.071	26.752 ± 0.536	0.060 ± 0.010	18.684 ± 0.279	0.306 ± 0.016	12.381 ± 0.641	0.254 ± 0.029
17A	1986.934 ± 28.960	1.018 ± 0.040	22.757 ± 0.361	907.637 ± 4.534	0.193 ± 0.004	0.981 ± 0.003	5.722 ± 0.043	10.434 ± 0.155	0.312 ± 0.025	25.957 ± 0.292	0.028 ± 0.003	37.509 ± 0.856	0.271 ± 0.003
17B	1074.230 ± 10.177	0.929 ± 0.080	20.201 ± 0.563	46.563 ± 1.241	0.201 ± 0.004	1.372 ± 0.029	6.886 ± 0.149	10.528 ± 0.375	0.259 ± 0.036	12.613 ± 0.289	0.050 ± 0.005	27.396 ± 1.547	0.310 ± 0.026
18A	823.658 ± 46.909	0.058 ± 0.003	95.120 ± 4.049	60.605 ± 2.389	0.419 ± 0.017	1.332 ± 0.091	4.206 ± 0.212	28.138 ± 1.170	0.016 ± 0.002	8.767 ± 0.254	0.279 ± 0.002	14.338 ± 0.541	0.297 ± 0.013
18B	929.809 ± 38.788	0.212 ± 0.024	114.597 ± 4.491	126.003 ± 4.726	1.316 ± 0.052	1.148 ± 0.044	3.752 ± 0.139	25.748 ± 0.992	0.089 ± 0.031	3.042 ± 0.194	0.553 ± 0.048	13.135 ± 0.463	0.597 ± 0.015
19A	6946.933 ± 54.792	0.140 ± 0.006	16.232 ± 0.157	141.052 ± 1.797	0.057 ± 0.001	1.983 ± 0.036	5.558 ± 0.078	16.799 ± 0.104	0.040 ± 0.004	81.301 ± 1.050	0.209 ± 0.007	88.232 ± 0.290	0.186 ± 0.008
19B	4513.306 ± 71.166	0.378 ± 0.055	11.022 ± 0.229	169.886 ± 3.151	0.149 ± 0.004	0.878 ± 0.010	5.808 ± 0.130	10.217 ± 0.241	0.088 ± 0.008	65.831 ± 0.992	0.368 ± 0.012	64.856 ± 2.517	0.335 ± 0.003
20A	2700.134 ± 59.234	2.117 ± 0.041	63.540 ± 0.761	811.384 ± 95.342	0.550 ± 0.016	1.715 ± 0.015	9.312 ± 0.264	96.704 ± 1.360	0.358 ± 0.009	139.026 ± 1.188	0.241 ± 0.012	242.423 ± 3.358	1.016 ± 0.041
20B	3445.670 ± 156.084	1.904 ± 0.172	40.656 ± 1.798	1096.383 ± 44.632	0.545 ± 0.020	1.845 ± 0.076	11.075 ± 0.515	47.975 ± 2.109	0.340 ± 0.015	124.275 ± 5.475	0.260 ± 0.021	224.239 ± 9.420	0.936 ± 0.073
21A	1366.154 ± 43.769	0.514 ± 0.013	44.822 ± 1.408	221.802 ± 7.389	0.191 ± 0.007	1.534 ± 0.029	3.215 ± 0.103	17.061 ± 0.786	0.123 ± 0.016	38.968 ± 0.611	0.298 ± 0.010	30.774 ± 0.760	0.354 ± 0.011
21B	1547.880 ± 9.708	0.686 ± 0.097	44.712 ± 0.743	288.343 ± 4.740	0.217 ± 0.003	1.756 ± 0.026	4.590 ± 0.058	17.014 ± 0.241	0.157 ± 0.008	37.018 ± 0.756	0.459 ± 0.008	47.359 ± 0.698	0.433 ± 0.014
22A	2783.652 ± 71.490	0.566 ± 0.012	14.662 ± 0.192	496.003 ± 9.724	0.108 ± 0.001	0.588 ± 0.029	12.014 ± 0.234	23.083 ± 0.229	0.127 ± 0.004	32.348 ± 0.426	0.038 ± 0.003	30.619 ± 0.520	0.125 ± 0.002
22B	2425.634 ± 69.990	1.736 ± 0.097	27.918 ± 0.794	976.087 ± 28.935	0.382 ± 0.013	1.835 ± 0.060	14.685 ± 0.467	31.563 ± 1.213	0.568 ± 0.030	34.086 ± 1.063	0.075 ± 0.005	38.591 ± 2.060	0.743 ± 0.027
23A	4875.047 ± 96.830	0.763 ± 0.027	20.947 ± 0.676	577.959 ± 12.425	0.175 ± 0.004	0.478 ± 0.017	8.750 ± 0.373	15.685 ± 0.419	0.190 ± 0.010	30.978 ± 1.054	0.018 ± 0.001	10.407 ± 0.459	0.145 ± 0.006
23B	3247.151 ± 229.628	0.839 ± 0.081	15.328 ± 1.275	319.558 ± 25.709	0.220 ± 0.016	0.751 ± 0.071	10.029 ± 0.844	14.596 ± 1.137	0.193 ± 0.019	24.580 ± 2.076	0.021 ± 0.002	9.349 ± 0.738	0.232 ± 0.018
24A	3624.597 ± 131.765	0.323 ± 0.015	21.411 ± 0.818	281.248 ± 13.346	0.157 ± 0.006	0.211 ± 0.018	6.712 ± 0.384	35.454 ± 0.716	0.204 ± 0.010	41.944 ± 1.497	0.016 ± 0.001	160.701 ± 5.207	0.435 ± 0.007
24B	1232.121 ± 13.303	0.651 ± 0.043	15.871 ± 0.341	226.296 ± 4.284	0.138 ± 0.003	0.373 ± 0.010	7.423 ± 0.115	36.031 ± 0.391	0.161 ± 0.026	24.846 ± 0.414	0.042 ± 0.006	41.682 ± 0.956	0.599 ± 0.028
25A	3486.329 ± 43.894	0.112 ± 0.008	10.634 ± 0.195	87.808 ± 1.330	0.068 ± 0.003	0.275 ± 0.016	3.259 ± 0.086	16.708 ± 0.595	0.138 ± 0.069	9.844 ± 0.353	0.014 ± 0.001	4.255 ± 0.112	0.101 ± 0.016
25B	2129.559 ± 74.299	0.055 ± 0.006	8.802 ± 0.336	60.527 ± 2.199	0.074 ± 0.003	0.541 ± 0.030	4.723 ± 0.200	17.704 ± 0.534	0.037 ± 0.009	17.240 ± 0.436	0.004 ± 0.001	2.122 ± 0.120	0.025 ± 0.002
26A	2845.393 ± 47.820	0.364 ± 0.006	54.371 ± 1.068	301.034 ± 3.983	0.112 ± 0.005	0.691 ± 0.007	8.105 ± 0.091	20.441 ± 0.593	0.049 ± 0.002	27.172 ± 0.448	0.198 ± 0.006	44.065 ± 0.814	0.365 ± 0.009
26B	1414.877 ± 37.451	0.919 ± 0.042	39.879 ± 0.852	324.953 ± 8.002	0.165 ± 0.006	1.047 ± 0.031	18.343 ± 0.617	18.046 ± 0.405	0.181 ± 0.016	19.245 ± 0.435	0.798 ± 0.037	34.710 ± 1.369	0.837 ± 0.038

Each measurement was performed in triplicate and the result is presented as the average ± SD. All values are in mg kg^−1^. Batches of herb roots from 2020 and 2021 are marked as A and B, respectively.

**Table 5:** Descriptive statistic of measurement results for 13 elements in 26 herbs each analyzed in two batches purchased 1 year apart ($n=52$).

	Mg	V	Mn	Fe	Co	Ni	Cu	Zn	As	Sr	Cd	Ba	Pb
Minimum	643	0.047	6.50	19	0.050	0.21	1.62	10.2	0.016	3.04	0.004	2.12	0.021
5th percentile	804	0.057	8.79	39	0.055	0.31	2.89	10.5	0.027	7.03	0.013	4.33	0.042
1st quartile	1234	0.294	13.3	173	0.113	0.59	4.62	16.7	0.085	17.4	0.037	9.25	0.126
Average	2187	0.845	46.2	416	0.285	1.17	6.94	25.5	0.213	41.6	0.150	43.2	0.686
Median	1937	0.612	20.8	310	0.187	0.98	6.23	20.2	0.169	27.2	0.067	22.8	0.284
IQR	2187	0.845	46.2	416	0.285	1.17	6.94	25.5	0.213	41.6	0.150	43.2	0.686
3rd quartile	2765	0.997	44.8	574	0.410	1.75	8.86	29.0	0.283	41.1	0.210	45.5	0.599
95th percentile	4955	2.403	206	1133	0.870	2.95	13.9	65.0	0.646	162	0.595	227	3.644
Maximum	6947	3.635	475	1538	1.316	3.08	18.3	119	0.865	261	0.798	242	9.062

All values are in mg kg^−1^.

The ranges of contents of determined elements, arranged by the minimum measured content and expressed in mg kg^−1^, are as follows (number of herb in parenthesis): Cd from 0.004 (25B, *Trichosanthis*) to 0.798 (26B, *Vladimiriae* *soulier*), As from 0.016 (18A, *Pseudostellariae*) to 0.865 (7B, *Codonopsis*), Pb from 0.021 (14A, *Paeoniae* *alba*) to 9.062 (12A, *Morindae* *officinalis*), V from 0.047 (14A, *Paeoniae* *alba*) to 3.635 (7B, *Codonopsis*), Co from 0.050 (16B, *Platycodi*) to 1.316 (18B, *Pseudostellariae*), Ni from 0.21 (24A, *Stephaniae* *tetrandrae*) to 3.08 (8B, *Dipsaci*), Cu from 1.62 (11B, *Isatidis*) to 18.3 (26B, *Vladimiriae* *soulier*), Ba from 2.12 (25B, *Trichosanthis*) to 242 (20A, *Pulsatillae* *chinensis*), Sr from 3.04 (18B, *Pseudostellariae*) to 261 (15B, *Paeoniae* *rubra*), Mn from 6.50 (10B, *Glehniae*) to 475 (12A, *Morindae* *officinalis*), Zn from 10.2 (19B, *Puerariae*) to 119 (7B, *Codonopsis*), Fe from 19 (14A, *Paeoniae* *alba*) to 1538 (7B, *Codonopsis*) and Mg from 643 (5B, *Asparagi*) to 6947 (19A, *Puerariae*). The largest content of four elements (As, V, Zn, Fe) were measured for herb 7B (*Codonopsis*).

The contents obtained in roots of herb 14 (*Paeoniae* *alba*) are close to those published by Xue et al. for the same plant species, which may suggest that the origin of both herbs or the cultivation conditions were similar^44^. The contents of elements causing toxic effects reported in the literature for the same herb species may vary greatly, for example for herb 4 (*Angelicae* *sinensis*), as presented by Fu et al.^29^: 2.616 mg kg^−1^ of As therein vs. 0.440–0.630 mg kg^−1^ in this paper, 8.107 mg kg^−1^ of Cd vs. 0.017–0.048 mg kg^−1^ and 11.428 mg kg^−1^ of Pb vs. 0.350–0.630 mg kg^−1^. The elevated content of elements in cultivated herbs may reflect the extent of pollution arising from overuse of artificial fertilizers and pesticides, improper disposal of industrial and residential wastes or vehicle exhaust^29^. In contrary, Filipiak-Szok et al. obtained values for *Angelicae* *sinensis* which closer correspond with our results: 0.38 mg kg^−1^ for As, 0.15 mg kg^−1^ for Cd 0.45 mg kg^−1^ for Pb, 0.95 mg kg^−1^ for Ni and 3.67 mg kg^−1^ for Ba^28^.

The ranges of content of determined elements in roots of herbs in this work do not generally exceed the minimum and maximum values found in literature. In review article Pohl et al. provided the ranges of contents of elements determined in herbal products by spectroscopic methods after acidic digestion gathered from 44 research articles with description of digestion procedures^43^. The ranges of contents of elements causing toxic effects in herbal products cited therein are sometimes very broad—over 3 orders of magnitude, e.g. 0.067–148 mg kg^−1^ for Ni (0.21–3.08 mg kg^−1^ in this study), 0.012–0.75 mg kg^−1^ for As (0.016–0.865 mg kg^−1^ in this study), 0.001–9.30 mg kg^−1^ for Cd (0.004–0.798 mg kg^−1^ in this study), 0.012–64.4 mg kg^−1^ for Pb (0.021–9.062 mg kg^−1^ in this study), 0.19–212 mg kg^−1^ for Sr (3.04–261 mg kg^−1^ in this study) and 1.40–181 mg kg^−1^ for Ba (2.12–242 mg kg^−1^ in this study).

The method of cultivation and exact geographical origin of herbs in this study is unknown, besides that they come from China. The differences between contents of elements in herbs in this study and the literature data may arise from differences in: method of cultivation, type of soil, pollution of air, water and soil, pH of soil and water, type and amounts of fertilizers, plant species, climatic condition, plant growth time and conditions, part of the plant, storage conditions and possibly more. Some regions of China are more exposed to discharge of wastewaters, solid and gas waste which are dumped to the environment as a result of mining and smelting industry, traffic, fertilization, soil amendment with sludge from wastewater treatment plants, inadequate irrigation practices which raise concerns regarding their impact on environment, plants cultivation and inhabiting people^3,45^. Gao et al. showed that soil from polluted areas and the herbs growing there are severely contaminated, in comparison to soils and herbs from unpolluted areas^3^.

According to WHO the maximum permissible amounts in dried plant material are 10 mg kg^−1^ of Pb and 0.3 mg kg^−1^ of Cd^46^. WHO did not propose limits for any other element, however, examples of national limits were proposed by several countries (for example Canada, China, Malaysia, Korea, Singapore, Thailand) and are within ranges: 2–5 mg kg^−1^ of As, 10–20 mg kg^−1^ of Pb, 0.3–1 mg kg^−1^ of Cd^47^. Considering the proposed limits, in most cases the maximum amounts of elements causing toxic effects is not exceeded in the tested roots of herbs. None of the investigated herb roots exceeded 10 mg kg^−1^ of Pb, and the maximum value of 9.1 mg kg^−1^ was measured for herb 12A (*Morindae* *officinalis*). In 7 herbs the content of Cd exceeded 0.3 mg kg^−1^ (13% of all tested samples) with the maximum value of 0.80 mg kg^−1^ for herb 26B (*Vladimiriae* *soulier*). The highest content of As—0.86 mg kg^−1^—was obtained for the herb 7B (*Codonopsis*) without exceeding the national limits.

The proper manner of preparing the medicinal herbs is the infusion, and the risk of introducing high amounts of harmful ingredients, including elements, is usually low due to the low extraction efficiency. However, the extraction efficiency varies substantially in different herbs and also different parts of plant and can be boosted in acidic conditions, i.e. by addition of lemon juice^48^. For black tea infusions the extractions efficiency of As, Cd, Pb, Ni, Sr and Ba were estimated as < 36.8%, < 40.3%, < 58.6%, 0.1–100%, 0.6–27.9% and 0.4–7.6%, respectively^49^. Wang et al. found that the extraction efficiency for Chinese herbal medicines was in range 6–62% for Cd, 0–8% for Pb and 1–15% for As^50^. The recommended dose of some herbal medicines is up to 30 g per single use and the patient may have been prescribed the multiple doses a day. The prolonged ingestion of elements causing toxic effects, especially As, Cd and Pb, daily through weeks or months may contribute to systemic and chronic toxicity. Although, the maximum permissible amounts per mass unit of dried herb in this study is not exceeded in most specimens, the use of larger amounts of herbal material to prepare the infusion will proportionally increase the amounts of ingested harmful ingredients. The herbal medicines may also be consumed directly as a functionalized, nourishing food known as medicine food homologous (MFH) which may considerably increase the intake of hazardous elements^29^.

The roots of herbs in this study were purchased in two terms 1 year apart and the results of both batches were taken into statistical analysis. For some herbs the differences between two batches were small, for example for herbs 20, 21, 10, 9, and for some were relatively large, for example for herbs 7, 1, 22, 3. The evaluation of complex measurement results for numerous elements and samples require the advanced multivariate statistical methods which will be discussed in detail in next chapters.

### Chemometric evaluation

The multielemental analysis of herbs provided a large dataset of 13 elements content in 52 samples. Such large datasets are difficult to interpret with basic statistics, so the multivariate methods were applied. The multivariate statistics allow to reduce the multidimensionality of data in order to find the most prominent relationships among the measured values for all tested herbs. The applied multivariate methods are: CVA based on interactions and interaction effects and multiple regression analysis.

#### Canonical variates analysis

The analysis of canonical variates was applied to show, simultaneously, the variability among all tested herb roots and between two herb roots of the same species, i.e. the same product purchased twice, 1 year apart. Some variability is expected, possibly due to the delivery of a completely different batch, but could also be the same batch that has been stored for a year in a storage facility. However, the content of the determined elements should not be expected to change after 1 year of storage of the herb roots, as it is a well-dried material and it is assumed that it was properly stored. The analyzed elements are not volatile and no losses during storage should occur. Drying is the standard way of preserving herbs which allow to keep the original characteristics and renders them available in any season^51^.

**Figure 1:**
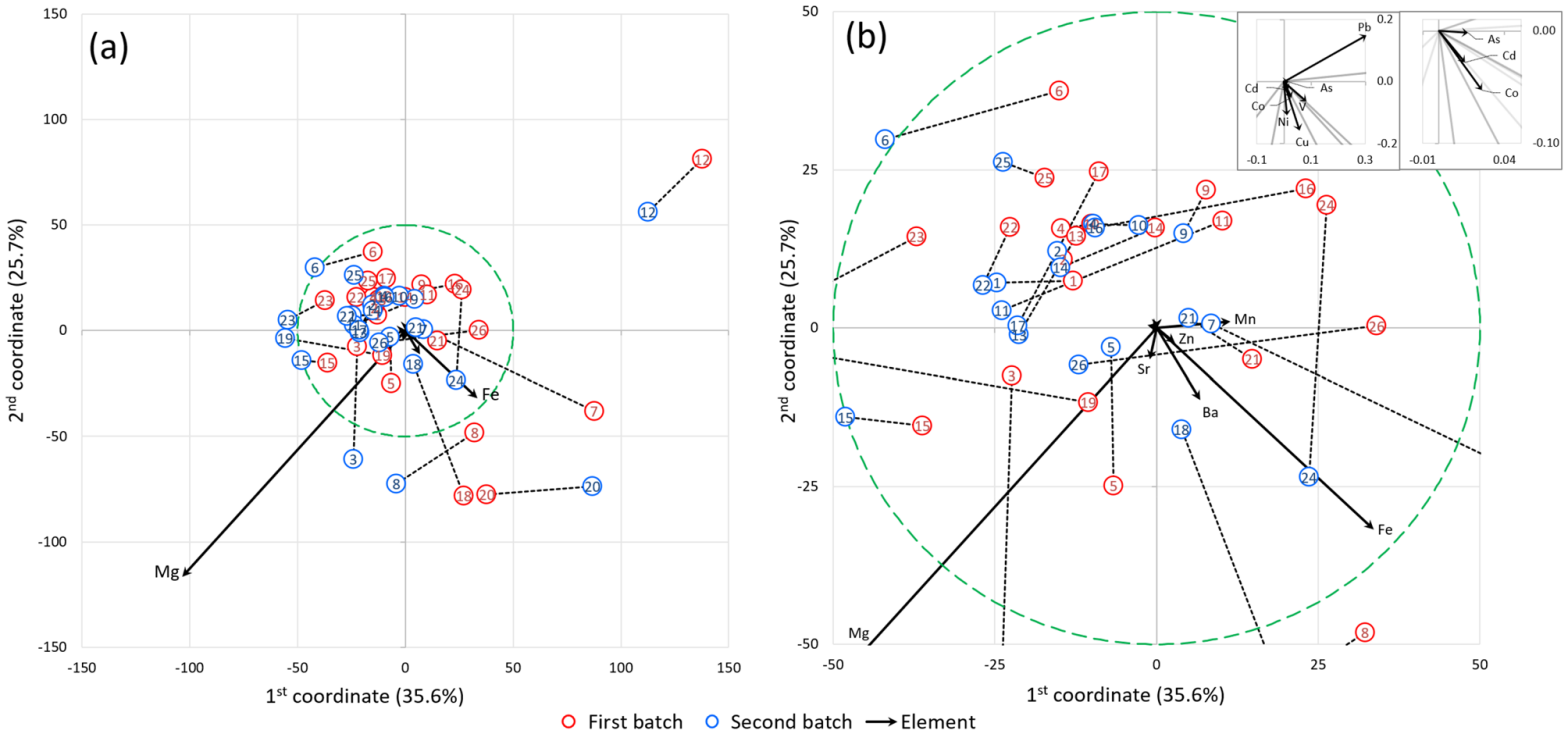
The results of CVA. Projection of objects on space defined by 1st and 2nd coordinate in relation to the elements in full scale (**a**) and zoomed section (**b**) outlined by the green dashed line. The two batches represent the herbs purchased 1 year apart. The points for the same herb root are connected by dashed black line. The zoomed section of the origin in upper right corner shows the direction of smallest arrows.

**Figure 2:**
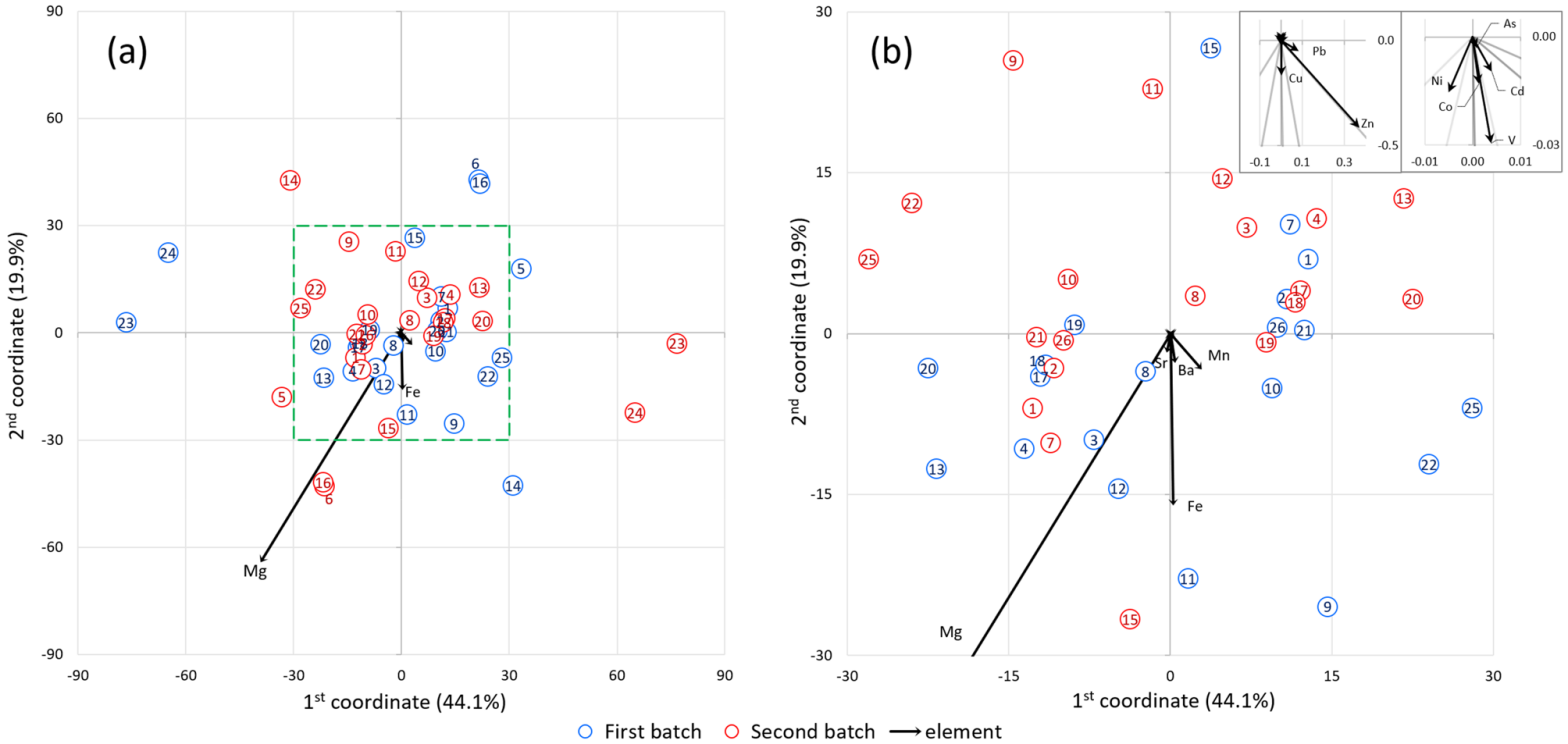
The projection of interaction effects. Projection of objects on space defined by 1st and 2nd coordinate in relation to the elements in full scale (**a**) and zoomed section (**b**) outlined by the green dashed line. The two data sets represent the herbs purchased 1 year apart. The points for the same herb roots from different batches are drawn in equal distances and on the opposite sides relative to the origin. The zoomed section of the origin in upper right corner shows the direction of smallest arrows related to elements.

Figure 1 shows what is the distribution of variability of concentrations of elements among two batches of herbs. The dimensionality reduction resulted in two coordinates which explain the 61.3% of total variability of determined elements in tested herb roots from two batches. The greater the variability, the longer the arrow. Dashed lines connect points that represent roots of herbs purchased in two terms. The length of this line indicates differences between two batches of the same herb. In terms of the content of elements, the herb roots differ substantially in the following years when the points for two batches are farther apart. The interaction effects are intended to show the effects of variables (type of herb and batch number) on the content of element and are presented in Fig. 2. The distance between the graph origin (the general mean) and the given point in graph (experimental object) is the Mahalanobis distance. The exact values of Mahalanobis distance for all points representing herbs from two batches presented in Figs. 1 and 2 are gathered in supplementary Table S1. The interaction effects for individual elements are presented in supplementary Fig. S1.

In some cases, the points corresponding to the two batches of roots of the same herb overlap or are very close together (see Fig. 1), for example 2, 4, 9, 10, 15, 25, so differences in element content between two batches are relatively small. The largest Mahalanobis distances between two points are for herbs 3, 7, 12, 18, 24. For those herbs the differences of content of element between two batches are relatively large. For most pairs of points, the direction of line connecting them is similar to the direction of arrows related to Mg and Mn, which indicates general differences in the content of Mg and Mn in the samples of the same herb from two batches. Magnesium is the most important in determining the position of points in this plane. Both batches of herbs 12 (*Morindae* *officinalis*) have relatively low Mg content compared to the rest of the samples. The points for the root 12 from both series differ significantly from the others on the chart (see Fig. 1). This is due the positive influence of Mn and Pb and the negative influence of Mg in the herb roots in both batches, as well as the largest contents of Pb and Mn of all tested herbs. The variability of several samples is largely determined by Fe and Ba: 20, 8, and only one from the series in the case of 7 and 18. Herb roots no. 7 (*Codonopsis*) differ significantly in the content of Fe, but also the content of other elements. The contents of the elements for the herb from the first batch are higher which determines their position along the arrows corresponding to Fe and Ba at a large distance from each other (see Fig. 1). In terms of intergroup relationships, Mn and Mg have the inverse relationship, while Ba and Fe have positive. A small relationship of Fe with Mn and Zn is observable, but the increase in Fe and Ba content has no significant effect on Mg content. Herb 7 shows relatively large differences of element content between two batches but the interaction effect is not large. And conversely, in herb 23 (*Scrophulariae*) the differences between two batches are not large (see Fig. 1), but the interaction effects graph show the considerable differences (see Fig. 2). The CVA analysis allowed to find the herb roots which are outliers in terms of content of elements and also helped to reject those results from the dataset resulting in the model of the regression analysis with $n=41$. The limiting distance form the origin of the graph that allowed to reject 20% of herb roots is outlined by green circle in Fig. 1.

The results presented in the interaction effects graph suggest that the data interpretation should not be done based solely on the average of tested samples (herb roots) or on the average of the batches purchased 1 year apart. The analysis of data should not be restricted only to the specific herb or to the specific batch. When analyzing the data of elemental content in numerous herbs gathered in batches one has to take into account the variability associated with different herbs in analyzed samples, as well as different batches, simultaneously. There are differences of content of elements in tested herb roots, as well as in two batches, and the changes of contents of elements are not predictable and dependent from each other. When the interaction effect is significant then the values of variables do not change proportionally. The CVA shows that by including the additional batch of herbs the data inference show more profound relationships among determined elements in roots of herbs and the conclusions of the experiment may be more deep and wide.

The CVA described in this study seems to be of practical use with handling the results of analysis as a continuous monitoring process of a product in specified time intervals. This is especially valid when the changes in product composition or other characteristics has impact on the effect for consumer and may pose risk for health when it does not meet the specification.

#### Regression analysis

**Figure 3:**
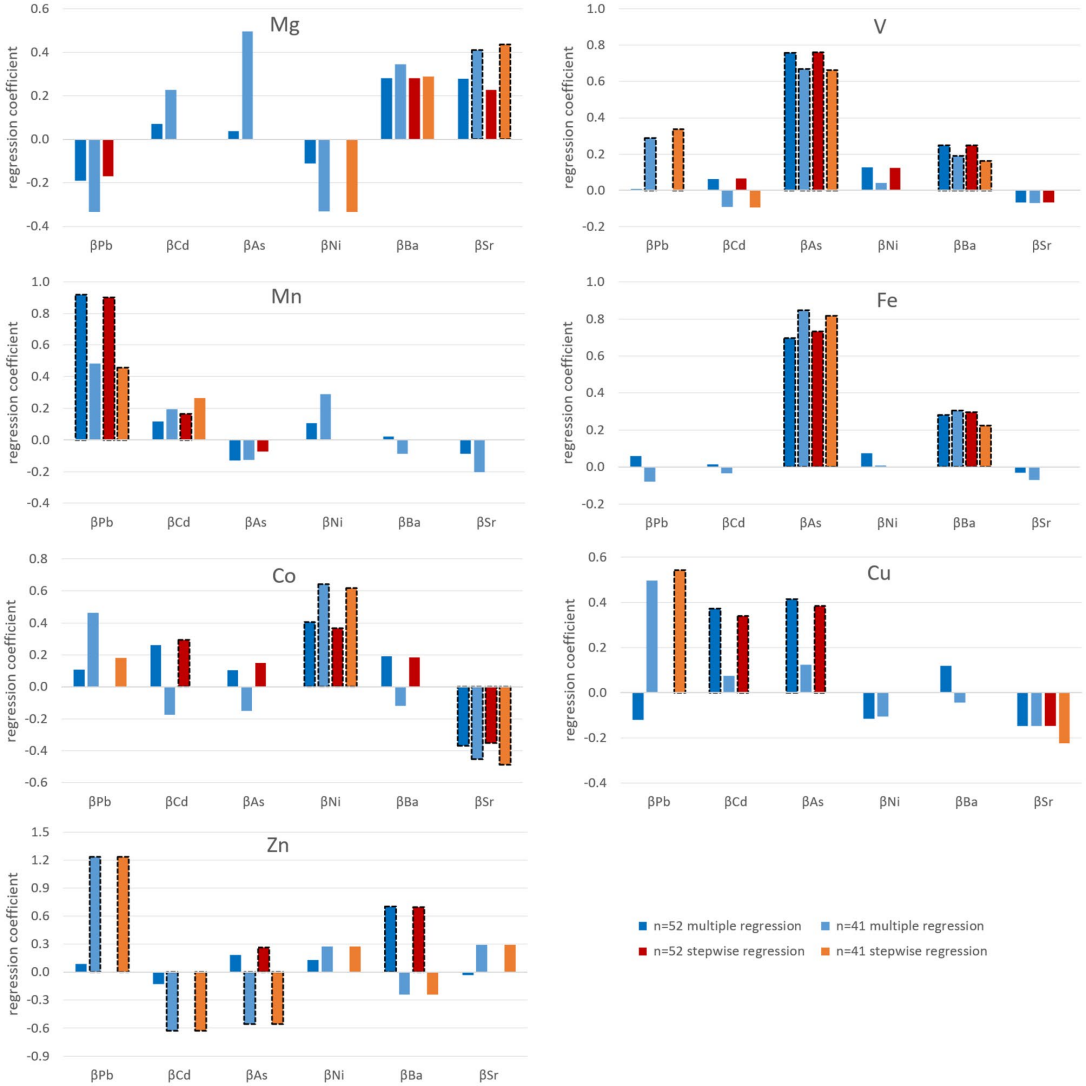
Comparison of regression coefficient β of four models of regression analysis: multiple regression ($n=52$ and $n=41$) and stepwise regression ($n=52$ and $n=41$) between physiological elements and elements causing toxic effects. The column with dashed outline represents the significant relationship.

The regression analysis was performed in order to find relationships between the determined elements in herb roots. The elements were divided into groups so that each element essential for humans was compared individually with a group of elements causing toxic effect. Two approaches to regression analysis, divided into 2 batches of samples of different sizes ($n=52$ and $n=41$), were taken and compared with each other resulting in four models of regression. Model $n=52$ contains all measurement results of 26 herbs from two batches. However, the CVA plot (see Fig. 1) indicates the presence of results that are far from the rest. The outlier values can significantly affect the relationships between the elements, so it was decided to check what effect the removal of outliers would have on the regression analysis. For this purpose, 20% of the analyzed herbs with the largest Mahalanobis distances, according to values in supplementary Table S.1 were rejected. As a result, 11 most outlier results were rejected, leaving 41 herbs form two batches which constituted the model $n=41$ in the regression analysis. The multiple regression shows how multiple variables, elements causing toxic effects, affect the physiological elements. Depending on the type of regression all elements causing toxic effects will be included in model or some of them will be excluded. The stepwise regression with backward elimination is also a multiple regression but some of the elements causing toxic effect are eliminated from the model, depending on the desired empirical significance level *p*, which in this study is 0.05. The results of 4 models of the regression analysis are presented in Fig. 3. Each physiological element is compared with elements causing toxic effect with the resulting regression coefficient. The column outlined by a dashed line shows the relation between two elements that is significant. The stepwise regression eliminates variables that are the least influencing to the total variation, therefore, the missing column may occur for 2 models: stepwise regression $n=52$ and $n=41$. The results of regression analysis are presented in supplementary Table S.2.

The calculated regression parameters for the four models show numerous similarities for some elements, which proves a significant relationship between them, regardless of the regression model. The multiple and stepwise regression show similar results but only in a specific group of samples: $n=52$ or $n=41$. The direction of relationship is concordant in most cases, as is presented by four models of regression in Fig. 3. The only difference in significance is visible for pairs of elements: Co–Cd and Zn–As for $n=52$ and Cu–Pb for $n=41$. Much pronounced differences are visible when comparing groups $n=52$ with $n=41$ where many differences are visible in graph. The most prominent are V-Pb, Mn-Pb, Co-Cd, Cu-Pb, Cu-Cd, Cu-As and all elements with Zn show more or less clear differences. Those elements have some non-standard or less predictable relationships, in comparison to other physiological elements. This suggests that the rejection of outliers, as is the case in group $n=41$, have much more notable impact on relationships between elements in studied samples than changing the type of regression from multiple to stepwise with backward elimination.

There are also several correlations which are significant for all 4 models of regression. Vanadium is correlated with arsenic ($0.66<\beta <0.76$) and with barium ($0.16<\beta <0.25$), Iron is correlated with arsenic ($0.69<\beta <0.85$), and cobalt is correlated with nickel ($0.36<\beta <0.65$) and negatively correlated with strontium ($-0.49<\beta <0.35$). There were samples with much higher content of certain element compared to the rest of samples, even over an order of magnitude. Such high concentration values in a single or few samples may have large effect on existing relationships between elements. The regression analysis in the model $n=41$ after removal of outliers showed relationships that did not exist or were not significant in the model $n=52$, e.g. Mg-Ni, V-Pb, Cu-Pb, and also Zn with all elements causing toxic effect. The elements causing toxic effects that have weak and the least significant relationships with physiological elements are Ni and Cd. Nickel is positively and significantly related only with Co, according to four regression models. The model $n=41$ shows that Cd is in inverse relationship with Zn, while according to the model $n=52$, Cd has positive relationship with Cu. Arsenic has strong and significant relationship with V and Fe. Nkansah et al. estimated the Pearson correlation coefficients for elements in black and green tea from Ghana and the significant and positive correlation were found for Zn-Cd ($r=0.625$) and Zn-As ($r=0.591$), however, in this study the stepwise regression ($n=41$) for those pairs showed also significant relationships, but in the opposite direction^31^.

## Conclusions

In this study the ICP-MS technique allowed to determine 13 elements, both physiological (Mg, V, Mn, Fe, Co, Cu, Zn) and causing toxic effects (Ni, As, Sr, Cd, Ba, Pb) in 26 roots of herbs originating from China which are used in TCM. The herbs were acquired from online shop in two batches 1 year apart to check the variation of elemental composition of the same product bought in time interval. The analytical procedure of sample preparation and analysis by ICP-MS was developed and validated. The use of ICP-MS with octopole reaction system and helium gas allowed to minimize the spectral interferences arising from matrix and argon gas. The application of matching-matrix CRMs allowed to estimate the parameters characterizing the performance of the analytical method and assured the traceability of measurement result. The multivariate statistical methods, such as the analysis of canonical variates and interaction effects, and multiple regression analysis allowed to find the relationships between the determined elements in roots of herbs, as well as between the two batches bought 1 year apparat. The obtained results showed the broad range of content of determined elements among all examined species of herbs.

The CVA allowed to show the magnitude of differences between two batches of herbs, taking into account each determined element and showing that for some herbs the differences were much more prominent than in others. The statistical inference should not be based solely on the type of herb or number of batch because of the underlying interaction effects between those two variables that may be a source of variability of the content of elements. The multiple regression analysis revealed the significant relationships between elements in all tested herb roots: Mg with Sr; V with Pb, As and Ba; Mn with Pb; Fe with As and Ba; Co with Ni and Sr, Cu with Pb, Cd and As; Zn with Pb, Cd, As and Ba.

The only element causing toxic effects that was found to excess the maximum permissible limit was Cd. The maximum permissible amount of Cd in dried plant material proposed by WHO is 0.3 mg kg^−1^ and this limit was exceeded in 7 herbs which makes 13% of all tested samples. No other element causing toxic effects was above the permissible limit, however, the prolonged consumption of roots of herbs and the infusions as the therapy in TCM may potentially be a cause of detrimental effects on health. Even if the maximum permissible limits are not reached, the recommended dose of certain herb roots can reach up to 30 g, and the multiple doses over a long time of therapy may constitute a considerable source of elements causing toxic effects like As, Cd, Pb or Ni that can develop chronic toxicity in organism.

## Supplementary Information

Supplementary Information.

## Data Availability

The original datasets described in this study are available on reasonable request from the corresponding author.
