# Supplementary material for: Chemometric approach to find relationships between physiological elements and elements causing toxic effects in herb roots by ICP-MS
Source: Sci Rep. 2021 Oct 19;11:20683. doi: 10.1038/s41598-021-00019-w (PMC8526606; doi:10.1038/s41598-021-00019-w)
Supplement: Supplementary file 1 — Supplementary Information. [file 41598_2021_19_MOESM1_ESM.docx]

SUPPLEMENTARY MATERIALS

Chemometric approach to find relationships between physiological elements and elements causing toxic effects in herb roots by ICP-MS

# Adam Sajnóg^1^*, Elwira Koko^1^, Dariusz Kayzer^2^, Danuta Barałkiewicz^1^

^1^Department of Trace Analysis, Faculty of Chemistry, Adam Mickiewicz University, Poznań, Uniwersytetu Poznańskiego 8, 61-614, Poznań, Poland.

^2^Department of Mathematical and Statistical Methods, Poznań University of Life Sciences, Wojska Polskiego 28, Poznań, 60-637, Poland.

* corresponding author, e-mail: [adam.sajnog@amu.edu.pl](mailto:adam.sajnog@amu.edu.pl)


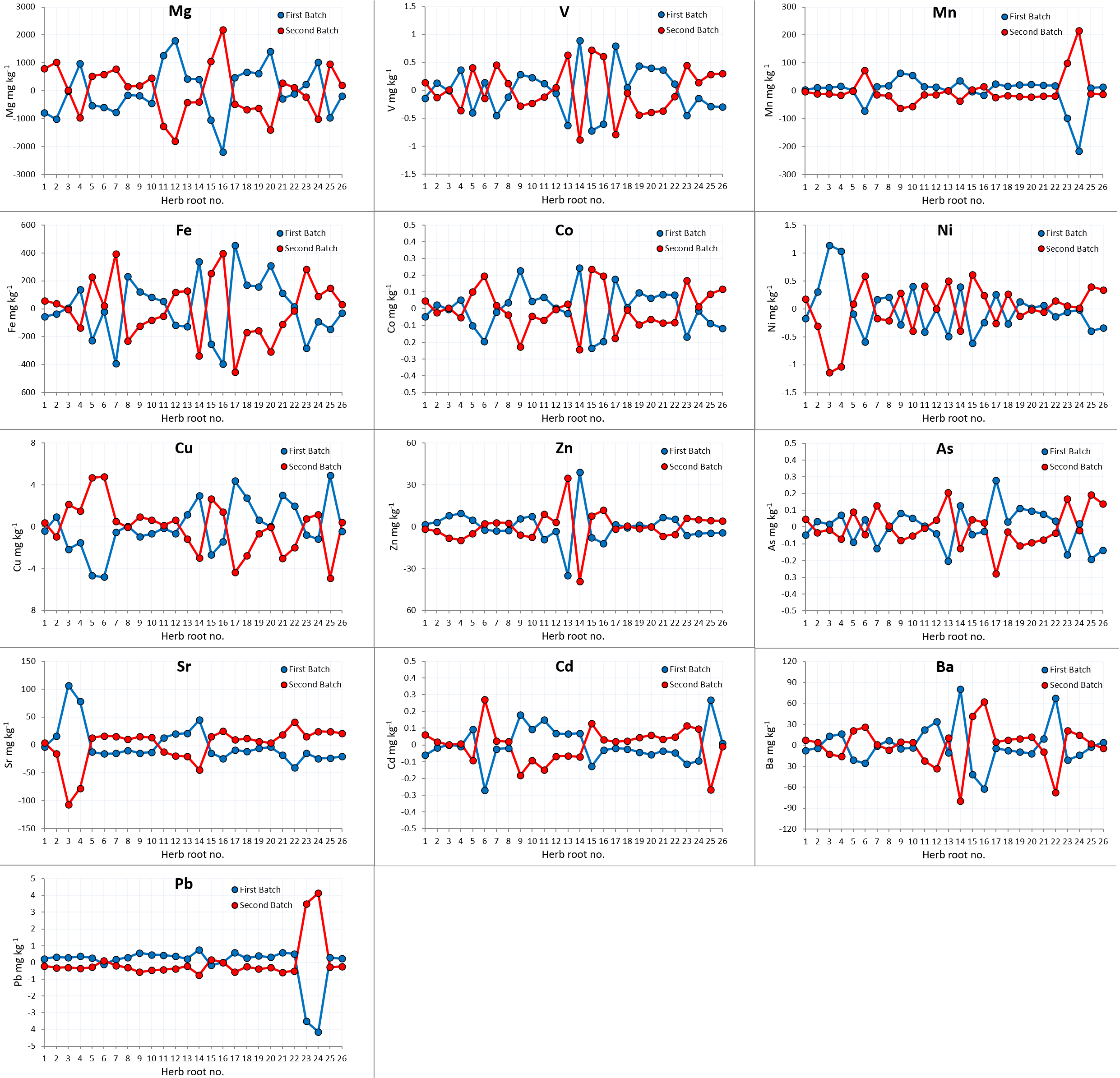


Supplementary Figure S1. The interaction effects for each element separately.

The more frequently two lines intersect, or the points switch side from positive to negative part of chart and *vice versa*, the more influence have the interaction effects on the variability of contents of elements in herb roots. The elements for which interaction effects do not substantially affect the variability are those where lines are often parallel and rarely intersecting, for example Pb, Sr, Mn or Zn. On the other hand, Mg, V, Fe and Ni show the large influence of interaction effects on the variability of elements in two batches of herb roots.

Supplementary Table S1. Mahalanobis distances between: experimental objects (herb roots) and the general mean (origin), and between the first and second batch. The below Mahalanobis distances are presented graphically in Fig. 1 and Fig. 2.

| Herb | Mahalanobis distance between | | | |
| --- | --- | --- | --- | --- |
|  | experimental object and the general mean | | first batch and second batch | |
|  | first batch | second batch | mean values | interaction effects |
| 1 | 30.00 | 19.95 | 19.62 | 15.24 |
| 2 | 22.89 | 22.99 | 12.81 | 14.99 |
| 3 | 93.86* | 37.55 | 84.68 | 41.17 |
| 4 | 31.26 | 44.03 | 18.59 | 33.61 |
| 5 | 29.08 | 64.23 | 41.85 | 41.96 |
| 6 | 56.85 | 45.35 | 33.60 | 54.92 |
| 7 | 24.58 | 111.72* | 108.27 | 23.74 |
| 8 | 80.49* | 69.20 | 63.41 | 11.83 |
| 9 | 22.67 | 30.69 | 12.72 | 43.68 |
| 10 | 24.76 | 26.36 | 8.41 | 27.28 |
| 11 | 39.36 | 30.52 | 50.64 | 27.14 |
| 12 | 149.62* | 163.45* | 90.60 | 27.87 |
| 13 | 34.51 | 39.10 | 23.96 | 38.47 |
| 14 | 26.36 | 25.39 | 19.28 | 59.88 |
| 15 | 79.71* | 90.75* | 31.86 | 29.97 |
| 16 | 22.71 | 44.70 | 40.61 | 49.35 |
| 17 | 29.97 | 35.51 | 47.38 | 31.83 |
| 18 | 48.28 | 117.82* | 84.00 | 21.10 |
| 19 | 78.30* | 38.86 | 56.00 | 19.46 |
| 20 | 129.68* | 103.28* | 57.76 | 33.20 |
| 21 | 29.22 | 38.84 | 15.53 | 19.29 |
| 22 | 37.33 | 45.58 | 33.01 | 43.25 |
| 23 | 63.06 | 44.75 | 28.09 | 80.06 |
| 24 | 74.88 | 40.37 | 68.18 | 76.05 |
| 25 | 39.29 | 32.60 | 13.59 | 39.16 |
| 26 | 24.75 | 59.92 | 65.15 | 17.25 |

*20 % of herbs with the largest Mahalanobis distances which were rejected in the models with *n*=41 of the regression analysis

Supplementary Table S2. Results of regression analysis between essential elements and a group of elements causing toxic effects: values of regression coefficients beta *β*, coefficient of determination *R*^2^ and probability *p*. Four models of regression were applied: multiple regression ($n=52$ and $n=41$) and stepwise regression with backwards elimination ($n=52$ and $n=41$). The regression coefficients that are significant are in red (*p*<0.05).

|  | Multiple regression | | Stepwise regression | |
| --- | --- | --- | --- | --- |
|  | *n*=52 | *n*=41 | *n*=52 | *n*=41 |
| Mg | | | | |
| *R*^2^ | 0.2159 | 0.2681 | 0.2093 | 0.2519 |
| *p* | 0.076 | 0.082 | 0.010 | 0.030 |
| *β*_Pb_ | -0.1913 | -0.3338 | -0.1685 |  |
| *β*_Cd_ | 0.0721 | 0.2266 |  |  |
| *β*_As_ | 0.0364 | 0.4956 |  |  |
| *β*_Ni_ | -0.1103 | -0.3317 |  | -0.3341 |
| *β*_Ba_ | 0.2819 | 0.3442 | 0.2811 | 0.2873 |
| *β*_Sr_ | 0.2783 | 0.4104 | 0.2259 | 0.4355 |
| V | | | | |
| *R*^2^ | 0.9038 | 0.9621 | 0.9037 | 0.9229 |
| *p* | <0.001 | <0.001 | <0.001 | <0.001 |
| *β*_Pb_ | 0.0070 | 0.2886 |  | 0.3382 |
| *β*_Cd_ | 0.0635 | -0.0921 | 0.0656 | -0.0929 |
| *β*_As_ | 0.7569 | 0.6697 | 0.7599 | 0.6622 |
| *β*_Ni_ | 0.1255 | 0.0419 | 0.1231 |  |
| *β*_Ba_ | 0.2496 | 0.1897 | 0.2491 | 0.1615 |
| *β*_Sr_ | -0.0672 | -0.0701 | -0.0662 |  |
| Mn | | | | |
| *R*^2^ | 0.8568 | 0.6992 | 0.8506 | 0.4405 |
| *p* | <0.001 | <0.001 | <0.001 | <0.001 |
| *β*_Pb_ | 0.9205 | 0.4826 | 0.8996 | 0.4579 |
| *β*_Cd_ | 0.1166 | 0.1930 | 0.1641 | 0.2657 |
| *β*_As_ | -0.1296 | -0.1272 | -0.0736 |  |
| *β*_Ni_ | 0.1056 | 0.2891 |  |  |
| *β*_Ba_ | 0.0233 | -0.0881 |  |  |
| *β*_Sr_ | -0.0866 | -0.2032 |  |  |
| Fe | | | | |
| *R*^2^ | 0.8051 | 0.7891 | 0.7987 | 0.7835 |
| *p* | <0.001 | <0.001 | <0.001 | <0.001 |
| *β*_Pb_ | 0.0580 | -0.0799 |  |  |
| *β*_Cd_ | 0.0134 | -0.0353 |  |  |
| *β*_As_ | 0.6953 | 0.8470 | 0.7329 | 0.8172 |
| *β*_Ni_ | 0.0735 | 0.0073 |  |  |
| *β*_Ba_ | 0.2823 | 0.3059 | 0.2960 | 0.2249 |
| *β*_Sr_ | -0.0318 | -0.0704 |  |  |

|  | | Multiple regression | | | Stepwise regression | | | |
| --- | --- | --- | --- | --- | --- | --- | --- | --- |
|  | | *n*=52 | | *n*=41 | *n*=52 | | | *n*=41 |
| Co | | | | | | | | |
| *R*^2^ | 0.4577 | | 0.5054 | | | 0.4483 | 0.4920 | |
| *p* | <0.001 | | <0.001 | | | <0.001 | <0.001 | |
| *β*_Pb_ | 0.1063 | | 0.4627 | | |  | 0.1825 | |
| *β*_Cd_ | 0.2619 | | -0.1758 | | | 0.2940 |  | |
| *β*_As_ | 0.1036 | | -0.1520 | | | 0.1503 |  | |
| *β*_Ni_ | 0.4044 | | 0.6416 | | | 0.3663 | 0.6192 | |
| *β*_Ba_ | 0.1908 | | -0.1196 | | | 0.1835 |  | |
| *β*_Sr_ | -0.3698 | | -0.4517 | | | -0.3532 | -0.4863 | |
| Cu | | | | | | | | |
| *R*^2^ | 0.3057 | | 0.3804 | | | 0.2815 | 0.3680 | |
| *p* | 0.009 | | 0.009 | | | 0.001 | <0.001 | |
| *β*_Pb_ | -0.1214 | | 0.4986 | | |  | 0.5432 | |
| *β*_Cd_ | 0.3736 | | 0.0747 | | | 0.3418 |  | |
| *β*_As_ | 0.4159 | | 0.1236 | | | 0.3863 |  | |
| *β*_Ni_ | -0.1166 | | -0.1053 | | |  |  | |
| *β*_Ba_ | 0.1203 | | -0.0438 | | |  |  | |
| *β*_Sr_ | -0.1481 | | -0.1488 | | | -0.1476 | -0.2234 | |
| Zn | | | | | | | | |
| *R*^2^ | 0.7160 | | 0.4492 | | | 0.6992 | 0.4492 | |
| *p* | <0.001 | | 0.002 | | | <0.001 | 0.002 | |
| *β*_Pb_ | 0.0906 | | 1.2331 | | |  | 1.2331 | |
| *β*_Cd_ | -0.1293 | | -0.6257 | | |  | -0.6257 | |
| *β*_As_ | 0.1843 | | -0.5531 | | | 0.2630 | -0.5531 | |
| *β*_Ni_ | 0.1278 | | 0.2762 | | |  | 0.2762 | |
| *β*_Ba_ | 0.7015 | | -0.2393 | | | 0.6952 | -0.2393 | |
| *β*_Sr_ | -0.0301 | | 0.2963 | | |  | 0.2963 | |
